# GeneXpert MTB/RIF assay in the diagnosis of urinary tuberculosis from urine specimens

**DOI:** 10.1038/s41598-017-06517-0

**Published:** 2017-07-21

**Authors:** Yu Pang, Yuanyuan Shang, Jie Lu, Qian Liang, Lingling Dong, Yunxu Li, Liping Zhao, Guanglu Jiang, Hairong Huang

**Affiliations:** 1 National Clinical Laboratory on Tuberculosis, Beijing Key laboratory for Drug-resistant Tuberculosis Research, Beijing Chest Hospital, Capital Medical University, Beijing Tuberculosis and Thoracic Tumor Institute, Beijing, China; 2 Beijing Pediatric Research Institute, Beijing Children’s Hospital, Capital Medical University, Beijing, China

## Abstract

Conventional bacteriological methods are not generally helpful in diagnosing urinary tuberculosis (UTB). GeneXpert is endorsed for the detection of pulmonary tuberculosis, whereas the data on its utility for urine specimens is limited. In this study, we aimed to evaluate its performance on urine specimens in a country with high TB incidence. A total of 163 suspected UTB patients were consecutively enrolled in the analysis, including 37 (22.7%) culture-positive and 44 (27.0%) clinically diagnosed UTB cases. Compared with conventional culture, the sensitivity of GeneXpert (94.6%) was significantly higher than that of smear microscopy (40.5%, *P* < 0.001). When setting clinical diagnosis as gold standard, 51 out of 81 clinically diagnosed UTB cases were detected by GeneXpert, demonstrating a sensitivity of 63.0%, which was significantly higher than that of smear microscopy (18.5%, *P* < 0.001) and culture (45.7%, *P* = 0.027), respectively. In addition, the proportion of UTB cases in the migrant population was significantly higher than that in the resident population (*P* = 0.019). To conclude, our data demonstrate that GeneXpert outperforms AFB smear and culture for the detection of MTB in urine samples, which provides an alternative for the diagnosis of UTB. The migrant population and previously diagnosed TB cases are high risk factors for developing UTB cases.

## Introduction

Tuberculosis (TB), caused by *Mycobacterium tuberculosis* complex (MTBC), remains a major global public health concern and is the first leading cause of death from infectious diseases worldwide^1, 2^. In 2015, an estimated 10.4 million people developed TB and 1.8 million died from the disease^1^. Most of the estimated number of cases occurred in Asia and African, and smaller proportions of cases occurred in European and American, especially in Latin America^1, 3, 4^. Of the 6.1 million notified incident cases, extrapulmonary TB (EPTB) represents 15% of global TB burden, ranging from 8% in the Western Pacific Region to 23% in the Eastern Mediterranean Region^1^. A series of studies from industrialized countries demonstrated that the contribution of EPTB to the total TB burden has significantly increased in recent years^5, 6^. Despite the increased trend seen in several regions, EPTB is rarely given high priority in the public health sector, which is mainly because EPTB is not significantly associated with the community transmission of the disease^5^. However, considering that EPTB contributes significantly to TB-related morbidity, severe complications and disabilities, there is an urgent need to address this group of patients in international TB control strategies^5, 7, 8^.

Urinary TB (UTB) is one of the most common types of EPTB, and is also considered as a severe form of EPTB in clinical practice^8–13^. About 20% of the EPTB cases reported annually are UTB^14^. The diagnosis of UTB is difficult because its symptoms are similar to other bacterial infections, which serves as the major cause for diagnosis delay and unfavorable treatment outcome of UTB patients^10^. The most important step in laboratory diagnosis of UTB is currently based on acid-fast staining and mycobacterial cultures^10, 15^. Acid-fast staining is cheap and fast but lacks sensitivity and reproducibility^16^. Cultures on media and liquid media yield an acceptable sensitivity, whereas the time-consuming procedures and long turn-around time cannot meet the criteria of point-of-care. Recently, GeneXpert MTB/RIF (GeneXpert), a fully automated real time hemi-nested PCR system, has been developed to rapid diagnosis of TB and rifampin (RIF) resistance in under 3 hours^17, 18^. The test utilizes five molecular beacons that detect mutations in an 81-bp core region of the *rpoB* gene that are associated with RIF resistance^19^. On the basis of numerous evaluation studies, World Health Organization (WHO) recommended this novel assay to diagnose pulmonary TB and RIF resistant in adults, as well as diagnose EPTB and RIF resistance in adults and children^20^. Unfortunately, given the limited data on the utility of GeneXpert for urine samples, these recommendations do not apply to these samples^20^. In this study, we have evaluated the performance of the GeneXpert on urine specimens for diagnosis of urinary TB in a country with high TB incidence.

## Materials and Methods

### Ethic statement

This study was approved by the Ethical Committee of Beijing Chest Hospital, Capital Medical University. The methods used in this study were performed in accordance with relevant guidelines and regulations. Each participant signed the informed consent prior to undergoing examination.

### Patient enrollment

A prospective study was conducted at Beijing Chest Hospital, a National Clinical Center of TB, between July 2015 and November 2016. A total of 167 patients with symptoms suggestive of UTB were enrolled in this evaluation. The clinical diagnosis of UTB was based on clinical symptoms, laboratory examinations and radiological signs. For the suspected patients without positive culture evidence, the clinical improvement after anti-TB treatment was considered as the only indicator for confirmed UTB cases, whereas the patients presenting no response after treatment were not considered as TB cases (Table 1). The demographic characteristics, including age, sex, residence, contact history and were collected from the medical records.

**Table 1:** Diagnostic criteria of urinary tuberculosis.

Classification	Definition
Definite urinary TB	Patients fulfill criterion A or B: A) Clinical suspected cases plus one or more of the following: positive smear microscopy examination; or positive mycobacterial culture examination. B) Clinical suspected cases plus positive pathological examination.
Clinically diagnosed urinary TB case	Clinical suspected cases plus the clinical improvement after empirical anti-TB treatment

### Laboratory examination

Each patient enrolled in this study provided one urine specimen, the volume of which was no less than 5 mL. Fluorescent smear microscopy was performed on all the specimens as described previously^21^. In addition, 2 mL of the samples were digested with N-acetyl-L-cysteine-NaOH-Na citrate (1.5% final concentration), and vortexed for 30 seconds. The treated urine samples were then incubated for 15 mins at room temperature, and then were neutralized with PBS buffer (pH = 7.4). After centrifugation at 4000 g for 15 min, the sediments were resuspended in 2 mL PBS buffer. 0.1 mL of resuspension was inoculated in the Lӧwenstein-Jensen (L-J) media. The tubes were incubated at 37 °C and monitored for mycobacterial growth for 8 weeks. Bacterial colonies were collected for conventional DST and species identification. The proportion method was used to detect the drug susceptibility of MTB isolates against RIF according to WHO recommendation. In addition, Tibilia rapid test, a commercial kit based on MPB64 antigen (Chuangxin, Hangzhou), was performed for species identification^22^.

For testing by GeneXpert (Cepheid, Sunnyvale, CA), 2 mL of each urine sample was mixed with 4 mL GeneXpert sample reagent, and incubated at room temperature for 15 min. Then 2 mL of digested sample was added to a GeneXpert cartridge and loaded onto the instrument. The results of the presence of MTB and RIF resistance were automated yielded by the instrument within approximately 2 hours.

### Statistical analysis

The performance of GeneXpert included the determination of sensitivity, specificity, positive predictive value (PPV) and negative predictive value (NPV). In addition, Chi-square test was used to compare the categorical variables in this study. *P* values of <0.05 were considered statistically significant. All the statistical analyses were performed with SPSS version 15.0 software (SPSS Inc., Chicago, IL, USA).

## Results

### Patients

**Figure 1:**
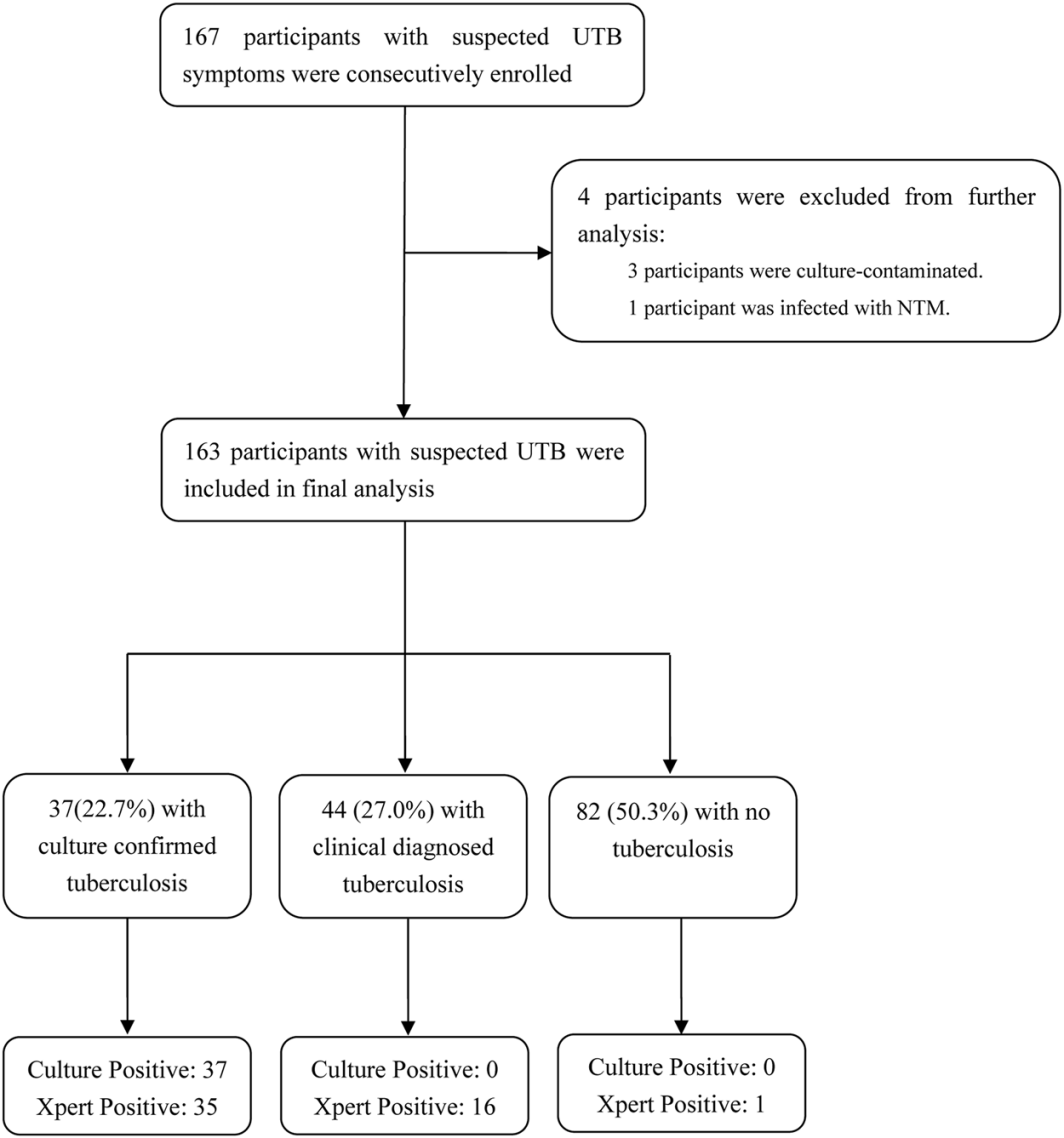
Enrollment of suspected urinary tuberculosis patients.

A total of 167 patients were consecutively enrolled in the study (Fig. 1). Of these patients, 4 (2.4%) were excluded from the study, since 3 (1.8%) were culture-contaminated and 1 (0.6%) were infected with nontuberculous mycobacteria (NTM); thus, the final sample size for the analysis were 163 participants. Among these 163 patients, 37 (22.7%) were culture-positive UTB cases, and 44 (27.0%) were diagnosed as UTB cases based on the clinical signs and outcomes of anti-TB therapy.

### Performance of GeneXpert for diagnosis of urinary tuberculosis from urine specimens

When compared with L-J culture, the sensitivity of acid-fast bacilli (AFB) microscopy and GeneXpert were 40.5% (15/37, 95% confidence interval [CI]: 24.7–56.4%) and 94.6% (35/37, 95% CI: 87.3–100.0%), respectively. In addition, GeneXpert identified 109 of 126 culture negative UTB cases, yielding a specificity of 86.5% (95% CI: 80.5–92.5%). Statistical analysis revealed that the sensitivity of GeneXpert was significantly higher than that of AFB microscopy (*P* < 0.001).

When setting clinical diagnosis as gold standard, the sensitivity and specificity of AFB smear were 18.5% (95% CI: 10.1–27.0%) and 98.8% (95% CI: 96.4–100.0%), respectively. The L-J culture identified twice as many cases as the AFB smear, the sensitivity and specificity of which were 45.7% (95% CI: 34.8–56.5%) and 100.0% (95% CI: 100.0–100.0%), respectively. As expected, the sensitivity of AFB smear was significant lower than that of L-J culture (*P* < 0.001). In addition, 51 out of 81 clinically diagnosed UTB cases were detected by the GeneXpert assay, demonstrating a sensitivity of 63.0% (95% CI: 52.4–73.5%), which was significantly higher than that of AFB smear (*P* < 0.001) and L-J culture (*P* = 0.027), respectively.

GeneXpert detected RIF resistance only in five patients, all of which were in accordance to phenotypic drug susceptibility testing, yielding a sensitivity of 100.0% (95% CI: 100.0–100.0%, Table 2).

**Table 2:** Performance of Xpert, AFB smear and solid culture for the diagnosis of urinary tuberculosis in urine samples.

Gold standard	Method	Sensitivity 95% CI	Specificity 95% CI	PPV^a^ 95% CI	NPV 95% CI
L-J culture	AFB smear	40.5% (15/37)^b^	99.2% (125/126)	93.8% (15/16)	85.0% (125/147)
		(24.7–56.4%)	(97.7–100.0%)	(81.9–100.0%)	(79.3–90.8%)
	GeneXpert	94.6% (35/37)	86.5% (109/126)	67.3% (35/52)	98.2% (109/111)
		(87.3–100.0%)	(80.5–92.5%)	(54.6–80.1%)	(95.7–100.0%)
Clinical diagnosis	AFB smear	18.5% (15/81)^c^	98.8% (81/82)	93.8% (15/16)	55.1% (81/147)
		(10.1–27.0%)	(96.4–100.0%)	(81.9–100.0%)	(47.1–63.1%)
	L-J culture	45.7% (37/81)	100.0% (82/82)	100.0% (37/37)	65.1% (82/126)
		(34.8–56.5%)	(100.0–100.0%)	(100.0–100.0%)	(56.8–73.4%)
	GeneXpert	63.0% (51/81)	98.8% (81/82)	98.1% (51/52)	73.0% (81/111)
		(52.4–73.5%)	(96.4–100.0%)	(94.3–100.0%)	(64.7–81.2%)
L-J DST	GeneXpert	100.0% (5/5)	100.0% (30/30)	100.0% (5/5)	100.0% (30/30)
		(100.0–100.0%)	(100.0–100.0%)	(100.0–100.0%)	(100.0–100.0%)

^a^PPV, positive predictive value; NPV, negative predictive value; AFB, acid-fast bacilli; L-J, Lӧwenstein-Jensen. ^b^L-J culture was set as gold standard: *χ* ^2^ = 24.67, *P* < 0.001 (AFB smear Se. vs. GeneXpert Se.). ^c^Clinical diagnosis was set as gold standard: *χ* ^2^ = 33.14, *P* < 0.001 (AFB smear Se. vs. Genexpert Se.); *χ* ^2^ = 13.71, *P* < 0.001 (AFB smear Se. vs. L-J culture Se.); *χ* ^2^ = 4.88, *P* = 0.027 (L-J culture Se. vs. GeneXpert Se).

### Demographic characteristics of UTB patients

We further compared the distribution of demographic characteristics between UTB cases and non-UTB cases. As shown in Table 3, the proportion of UTB cases in the migrant population, who had sought health care from other provinces of China, was significantly higher than that in the resident population (OR [95% CI]: 2.29 [1.14–4.59], *P* = 0.019), indicating that the migrant population was a risk factor for developing UTB cases. As expected, the distribution of patients previously diagnosed as TB in the UTB case group was significantly higher than that of patients without previous TB history (OR [95% CI]: 15.78 [5.78–43.08], *P* < 0.001). In contrast, we observed no statistical difference in the distribution of the UTB cases among different sex and age groups (*P* > 0.05).

**Table 3:** Demographic Characteristics of clinical diagnosed urinary tuberculosis patients enrolled in this study.

Characteristics	Diagnostic Class				
	UTB cases (81) N (%)	Non-UTB cases (82) N (%)	Odds ratios (95% CI)	*P* value	Total (163) N (%)
Sex					
Male	22 (27.2)	29 (35.4)	0.68 (0.35–1.33)	0.259	51 (31.3)
Female	59 (72.8)	53 (64.6)	1	—	112 (68.7)
Age group (years)					
<25	2 (2.5)	5 (6.1)	0.37 (0.07–2.02)	0.432	7 (4.3)
25~44	38 (46.9)	35 (42.7)	1	—	73 (44.8)
45~64	27 (33.3)	26 (31.7)	0.96 (0.47–1.94)	0.902	53 (32.5)
≥ 65	14 (17.3)	16 (19.5)	0.81 (0.34–1.89)	0.619	30 (18.4)
Population					
Resident	17 (21.0)	31 (37.8)	1	—	48 (29.4)
Migrant	64 (79.0)	51 (62.2)	2.29 (1.14–4.59)	0.019	115 (70.6)
TB history					
No	40 (49.4)	77 (93.9)	1	—	46 (28.2)
Yes	41 (50.6)	5 (6.1)	15.78 (5.78–43.08)	<0.001	117 (71.8)

UTB: urinary tuberculosis.

## Discussion

Due to the non-specific symptoms in the UTB patients, the diagnosis of this disease is still a major challenge worldwide, especially for the resource-limited settings^10^. In view of the inherent shortcomings of conventional diagnostic tests, the importance of a rapid, sensitive and highly specific diagnostic tool is urgently needed^10, 15, 23^. Here, we have evaluated the fully automated GeneXpert assay for diagnosis of UTB from urine specimens, and compared it with AFB smears as well as the L-J culture. Our data have demonstrated that GeneXpert outperforms AFB smear and solid culture for the detection of MTB in the urine samples. There is no doubt that the sensitivity of diagnostic tool depends on its limit of detection (LOD). Of the three methods, AFB smear require 5 × 10^3^ to 1 × 10^4^ bacilli/ml of specimen to yield a positive result^24^, whereas the LOD of GeneXpert assay is reported to be 131 CFU/ml of specimen^19^. For mycobacterial culture, despite exhibiting low LOD in the previous report^24^, its sensitivity was not comparable to that of GeneXpert. We hypothesize that the sensitivity of GeneXpert might have been enhanced by the detection of remnant DNA from dead bacterial cells. In addition to the poor LOD, the non-viscous nature of urine serves as an important contributor that weakening the fixation of bacilli on the smear, thus decreasing the positivity rate of AFB smear in urine samples^25^. Another limitation of AFB smear is that the possible presence of NTM will lead to “false positive” result. Despite these disadvantages, AFB smear is still a cost-effective screening tool for EPTB, especially in health settings with high TB burden^26^.

A previous study has reported that the culture of concentrated specimens can detect low concentrations of 100 bacilli/mL organisms^24^. The GeneXpert assay, in spite of sharing the similar LOD relative to the culture, exhibits better sensitivity in the diagnosis of MTB from the urine specimens. Compared with the high viscosity of sputum samples, the urine specimens are more homogeneous. In light of the same digested condition used in the treatment procedure for both sputum and urine samples, we hypothesize that this treatment procedure appears suitable for sputum, but too rigorous for urine. Hence, one important explanation for our observation may be due to the overexposure to the extreme alkaline environment of tubercle bacilli in the urine samples, which is responsible for inactivating a percentage of MTB, thereby resulting in the low recovery rate by conventional culture method^27^. Considering the findings in this study, further study is required to explore the optimal digested condition for urine samples, as well as other non-sputum samples.

Despite being an excellent technology for detection of UTB from urinary specimens, a major issue facing developing countries wishing to implement GeneXpert is the high cost compared to smear microscopy^28^. On the basis of our experience, the cost of GeneXpert is about 14 times higher than that of smear microscopy per patient in hospital sector (95 versus 6.5 US dollars), which could not enjoy the FIND-negotiated substantial price for use in the public sector. Therefore, the subsequent detection of MTB from smear-negative urinary specimens with GeneXpert may yield the optimal cost-effectiveness of GeneXpert testing.

Lipoarabinomannan (LAM), a glycolipid component of MTB cell wall, has been used as a promising marker for diagnosing active TB^29^. Recently, several commercial kits are developed for TB by detection of urinary LAM released from bacterial cells^30^. Several studies found that performance of urinary LAM in unselected TB suspects is unsatisfactory, whereas its diagnostic performance is significantly improved in HIV-infected patients^30^. One possible explanation for their favorable performance among HIV-infected TB patients may be due to the renal dysfunction associated with advanced HIV infection^30^. As a consequence, these novel kits seem only suitable for patients who live in settings with high prevalence of HIV. However, in view of the high frequency of the renal dysfunction due to urinary TB, LAM has the theoretical potential to be an attractive diagnostic option for UTB. Further evaluation will be carried out to assess the performance of LAM assay in detecting UTB in clinical practice.

Migrant population is a special group along with rapid economic development and urbanization in China^31^. Several previous studies announce that migrants are considered as a high-risk group for TB infection^31, 32^. Consistent to these findings, our results revealed that migrant population is at high risk for developing UTB disease. On one hand, due to unsatisfactory circumstances faced by the migrant population, this population suffers from lack of social assistance system, poor housing and public infrastructure, which increase health risk of TB incidence^31^. On the other hand, the unbalanced distribution of medical health resource in China contributes the transregional movement of UTB patients for seeking better health care service in the first-tier cities^33^. Although there is no evidence to confirm the transmission of UTB in the community, the diagnosis delay will lead to the poor clinical outcome, especially for this severe form of EPTB. In addition, we also identified that previously diagnosed TB cases are associated with increased risk for UTB. This is an understandable result as it has been shown that the UTB is usually caused by spread of tubercle bacilli through the blood stream since the initial infection, and the incubation period of MTB takes several years^10^. Therefore, the previously diagnosed TB accelerates the dissemination of active tubercle bacilli in the host, contributing the high prevalence of UTB in this population.

There were several obvious limitations in our study. First, due to low incidence of UTB, the major limitation of this study was the small sample size. Second, the conventional L-J media rather than liquid culture was used in this study, which may underestimate the detection sensitivity of culture method for MTB from urine specimens. Third, HIV infection is a risk factor for both pulmonary TB and EPTB. Because of the low prevalence of HIV infection in China, the HIV examination is not routinely detected among suspected TB cases. On the basis of our findings, the excellent performance of GeneXpert for detecting MTB in the urine samples need be verified by another study with sufficiently large sample size.

In conclusion, our data demonstrate that GeneXpert outperforms AFB smear and culture for the detection of MTB in the urine samples, which provides an alternative for the diagnosis of UTB. In addition, the migrant population and previously diagnosed TB cases are high risk factors for developing UTB cases in China.
